# Sodium‐Ion Batteries: Improving the Rate Capability of 3D Interconnected Carbon Nanofibers Thin Film by Boron, Nitrogen Dual‐Doping

**DOI:** 10.1002/advs.201600468

**Published:** 2017-01-20

**Authors:** Min Wang, Yang Yang, Zhenzhong Yang, Lin Gu, Qianwang Chen, Yan Yu

**Affiliations:** ^1^ CAS Key Laboratory of Materials for Energy ConversionDepartment of Materials Science and EngineeringUniversity of Science and Technology of ChinaHefeiAnhui230026China; ^2^ Hefei National Laboratory for Physical Sciences at Microscale and Department of Materials Science & EngineeringUniversity of Science and Technology of ChinaHefei230026China; ^3^ Beijing National Laboratory for Condensed Matter PhysicsThe Institute of PhysicsChinese Academy of SciencesBeijing100190China; ^4^ Collaborative Innovation Center of Quantum MatterBeijing100190China; ^5^ State Key Laboratory of Fire ScienceUniversity of Science and Technology of ChinaHefeiAnhui230026China

**Keywords:** 3D interconnected carbon, anode, Na‐ion batteries, rate capability

## Abstract

**Boron, nitrogen dual‐doping 3D hard carbon nanofibers thin film** is synthesized using a facile process. The nanofibers exhibit high specific capacity and remarkable high‐rate capability due to the synergistic effect of 3D porous structure, large surface area, and enlarged carbon layer spacing, and the B, N codoping‐induced defects.

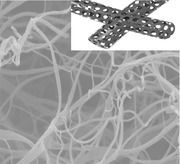

Over the last few years, Na‐ion batteries (NIBs) have attracted more and more attention as a low‐cost alternative energy system to Li‐ion batteries (LIBs), because Na is far more abundant in the earth's crust than Li and NIBs are environmental friendly for large‐scale energy storage (such as solar cell, wind power, and smart grid).^1^, ^2^ However, the progress of NIBs is slow because there are only few appropriate active materials with long cycle life and high‐rate capability for NIBs. Since the radius of Na^+^ is ≈55% larger than that of Li^+^, most materials do not possess host frameworks with larger interstitial spaces for Na‐ions insertion/extraction.^2^, ^3^ Most of the recent researches on the NIBs electrode material have focused mainly on the development of cathode.^1^, ^4^, ^5^, ^6^, ^7^ Graphite, the most commonly used anode in LIBs, cannot be used as the anode electrode material of NIBs due to the interlayer distance of graphite is too small to accommodate the larger Na^+^.^8^ Nongraphitic carbon materials are considered as the most promising candidates for NIBs anodes. Recently, a variety of carbonaceous materials, such as carbon with different morphologies (carbon nanowire,^9^ porous carbon,^10^ hollow carbon nanosphere,^11^ and layered reduced graphene oxides^12^), different composition (phosphorene–graphene^2^), and different structures (soft carbon and hard carbon), have been investigated as anodes for NIBs. However, most of the satisfied capacities of carbonaceous materials are obtained at low current density, indicating the sluggish kinetics for the sodium storage process.

To achieve high‐rate capability of NIBs, it is necessary to construct an electrode with decreased diffusion distance for ions, high Na^+^ diffusion coefficients, and fast electrons transport, which transports both electrons and Na^+^ quickly to the active materials (or active interfaces).^13^ The first popular approach to decrease the Na^+^ diffusion distance is to downsize the particle size as the diffusion time is proportional to *D*_Na_/(particle size)^2^ (here *D*_Na_ represents the sodium diffusion coefficients in solid).^14^ In addition, to achieve fast electrons transport, the most effective way is to make the material with high electronic conductivity and hierarchically porous structures.^15^, ^16^, ^17^, ^18^ Guo and co‐workers have demonstrated that a sandwich‐like hierarchically porous carbon/graphene nanocomposite^10^ delivered a high specific capacity of 250 mAh g^−1^ at a current rate of 1 A g^−1^ over 1000 cycles.

Recently, the engineering of carbonaceous nanomaterials by heteroatoms doping (i.e., nitrogen (N), boron (B), sulfur (S), phosphorus (P), iodine (I) doping) has attracted more and more attention, because it is an effective way to tune the electrical performance of their surface and improve their energy storage performance.^19^, ^20^, ^21^, ^22^, ^23^, ^24^, ^25^, ^26^ Among them, nitrogen (N) is the most extensively studied heteroatom, which could improve the reactivity and electronic conductivity by creating extrinsic defects.^27^, ^28^ Boron (B) doping is also the essential modified approach of carbon‐based materials, creating many active sites, and enhancing the conductivity of carbon materials obviously.^26^, ^29^ Compare to single‐atom doping, dual‐atom doping in carbon could exploit synergistic effects of the beneficial influences of the two heteroatoms.^30^ So it is a promising option to codoping carbon with two different heteroatoms, which takes advantage of the additionally created defect sites and electronic conductivity for sodium storage.^31^ To further increase the energy density and power density of the battery, metal current collector and binder are not suggested to use, because these materials could not participate in sodium/lithium storage.^32^

Herein, to combine all abovementioned approaches, we designed a self‐supported boron (B), nitrogen (N) codoping 3D interconnected carbon nanofibers thin film (denoted as BN‐CNFs) that displayed superior sodium storage performance. We developed a facile and sustainable approach for large‐scale production of BN‐CNFs by fully infiltrate NH_4_HB_4_O_7_·H_2_O into the bacterial cellulose (BC) pellicle followed by carbonization. Our approach has the following merits for constructing dual‐atom doping in 3D interconnected carbon materials: (i) The fabrication process is facile, simple, and easy scale‐up;^33^ (ii) The raw materials are abundant, cheap, and environment friendly.^34^ The BN‐CNFs could be used as electrode for NIB directly. It exhibits a high reversible charge capacity (691 mAh g^−1^ at 100 mA g^−1^) and superior rate capability 277 mAh g^−1^ after 1000 cycles at 10 A g^−1^). The special architecture and composite of BN‐CNFs provides a perfect solution hitting various birds with one stone, resulting in the outstanding sodium storage performance. First, the BN‐CNFs possess porous and 3D interconnected structure that facilitates the migration of Na^+^, leading to superior rate capability.^31^ Second, dual‐atom doping not only creates defect sites in CNFs but also improves the electrochemical activity and electronic conductivity.^30^ Finally, the robust 3D interconnected BN‐CNFs could accommodate the volume change during Na^+^ repeated insertion/extraction, ensuring the long cycle life.

**Scheme** **1** shows the detailed preparation process of BN‐CNFs electrode. First, the BC was immersed into an aqueous solution of NH_4_HB_4_O_7_·H_2_O for 24 h at room temperature. Next, the sample was freeze‐dried 24 h followed by carbonization in argon (Ar) atmosphere, leading to the in situ formation of porous BN‐CNFs. The amount of heteroatom doping could be adjusted by tuning the concentration of NH_4_HB_4_O_7_ **·**H_2_O. For comparison, 0, 0.02, 0.1, and 0.15 m NH_4_HB_4_O_7_ **·**H_2_O was used, and the corresponding samples were denoted as CNFs, BN‐CNFs‐1, BN‐CNFs, and BN‐CNFs‐2, respectively.

**Scheme 1:**
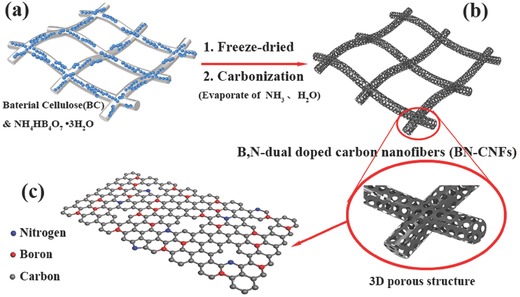
Fabrication process of BN‐CNFs electrode.

The morphologies of all samples were investigated by field emission scanning electron microscopy (FESEM) and transmission electron microscopy (TEM), as shown in **Figure** **1**. The BC treated with NH_4_HB_4_O_7_ **·**H_2_O solution (Figure 1a) exhibits interconnected fibrous morphology with a diameter of 80–100 nm. After a pyrolysis treatment, the obtained BN‐CNFs (Figure 1b) display a porous, cross‐linked, and well‐organized 3D network structure. The diameter of BN‐CNFs is smaller (30–80 nm) than that of BC, which is due to evaporation of CO, CO_2_, methanol, and acetic acid during the carbonization process.^35^ The TEM image of BN‐CNFs (Figure 1c) confirms its porous structure, enabling fast electron and ion transport along 3D directions. The high‐resolution transmission electron microscopy (HRTEM) image of the BN‐CNFs (Figure 1d) reveals a disordered pattern with only localized short‐range ordering. The interlayer spacing of (*d*_002_) is measured to be 0.44 nm, which importantly in the context of Na^+^ insertion, is significantly larger than that in graphite (0.34 nm).^11^, ^36^ Figure S1 (Supporting Information) reveals that the comparative products (CNFs, BN‐CNFs‐1, and BN‐CNFs‐2) exhibit similar morphology with the BN‐CNFs. The average interlayer distance of (*d*_002_) is measured to be ≈0.42 nm for CNFs, 0.46 nm for BN‐CNFs‐1 and 0.52 nm for BN‐CNFs‐2, respectively, indicating that the distance between carbon layers is expanded by doping the B and N atoms into carbon. The occurrence of large free space between hard carbon layers should be beneficial for the reversible storage of Na^+^.

**Figure 1:**
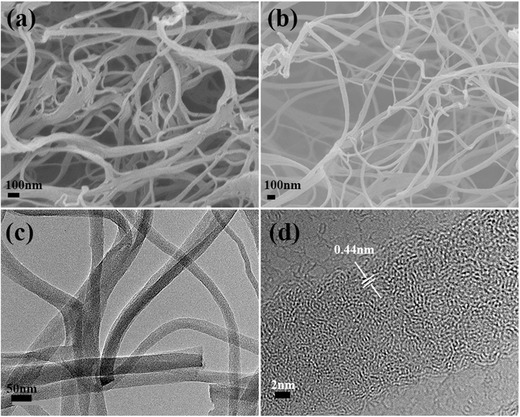
FESEM images of a) BC treated with NH_4_HB_4_O_7_ **·**H_2_O solution and b) the BN‐CNFs. c) TEM images of BN‐CNFs. d) HRTEM image of BN‐CNFs.

**Figure** **2**a shows the X‐ray diffraction (XRD) patterns of all the prepared samples. An obviously broadened diffraction peak can be observed, corresponding to the (002) diffraction of the graphitic layer‐by‐layer structure. Figure 2b displays the disordered feature of all the obtained specimen by the Raman spectroscopy analysis, which exhibits the two prominent peaks at 1352 and 1575 cm^−1^ assigned to the D mode and the G mode, respectively. The intensity ratio of *I*_D_/*I*_G_ is 0.89 for CNFs, 0.92 for BN‐CNFs‐1, 0.96 for BN‐CNFs, and 0.98 for BN‐CNFs‐2. The higher doping amount of B, N in CNFs results in an increased the *I*_D_/*I*_G_ ratio, leading to the disorder structure.^2^, ^37^

**Figure 2:**
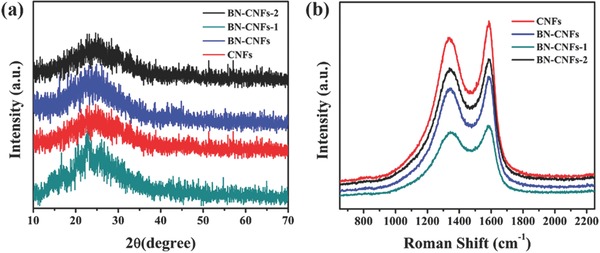
a) XRD patterns and b) Raman spectra of CNFs, BN‐CNFs‐1, BN‐CNFs, and BN‐CNFs‐2.

The specific surface area and pore structure of all samples were further characterized by nitrogen adsorption–desorption isotherms (**Figure** **3** and Figure S2, Supporting Information). Figure 3a exhibits an obvious steep increase in adsorption amount at very low relative pressure due to micropores filling.^38^ And the pronounced hysteresis loop reveal more developed mesopores. A growing number of mesopores are displayed in the pore size distribution curves of all samples as the amount of NH_4_HB_4_O_7_ **·**H_2_O increases as a result of harsher activation resulting in relatively larger pores.^39^ The Brunauer–Emmett–Teller (BET) specific surface area is 741 m^2^ g^−1^ for CNFs, 1280 m^2^ g^−1^ for BN‐CNFs‐1, 1585 m^2^ g^−1^ for BN‐CNFs, and 1556 m^2^ g^−1^ for BN‐CNFs‐2, respectively. Therefore, the BN‐CNFs have the highest BET surface area and a narrow pore size distribution compared to the other samples.

**Figure 3:**
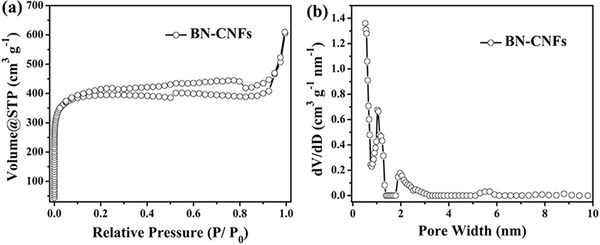
a) Nitrogen adsorption/desorption isotherms and b) the corresponding pore size distribution curve from the adsorption isotherms using density functional theory (DFT) method of the BN‐CNFs composite.

To analysis the B, N‐doping in carbon layer, X‐ray photoemission spectroscopy (XPS) measurement was carried out. The chemical compositions of all samples were summarized in Table S1 (Supporting Information). The reason for high B doping level would be explained in the Experimental Section. **Figure** **4**a,b shows the high‐resolution spectra of N 1s for the BN‐CNFs that are deconvoluted into four peaks corresponding to pyridinic N (398.8 eV), pyrrolic N (401.1 eV), quaternary N (402.1 eV), and C—N—B (399.2 eV).^27^, ^40^ The B_1s_ peak can be fitted to two peaks of BCO_2_ (192.3 eV)^41^ and N—B (191.0 eV).^42^ For BN‐CNFs‐2, when the amount of NH_4_HB_4_O_7_·H_2_O increases, the B concentration reduces (Table S1, Supporting Information). This is attributed to some carbon fibers crack due to the more severe carbonization reaction (Figure S3, Supporting Information), which would break the B—C—O bonds.^42^, ^43^ The elemental mapping (Figure 4c) of C, N, and B obviously demonstrates the N and B were homogeneously distributed in BN‐CNFs sample.^2^, ^44^

**Figure 4:**
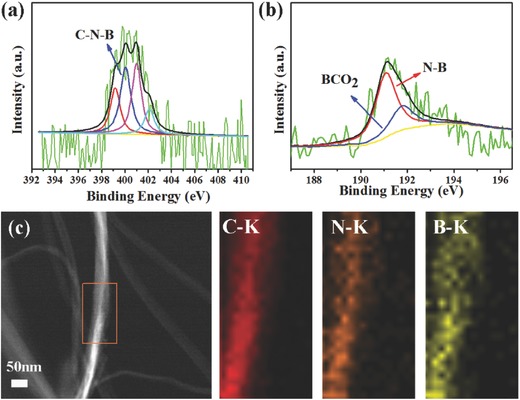
High‐resolution XPS a) N1s and b) B1s spectra for the BN‐CNFs sample. c) Elemental mapping images of BN‐CNFs electrode.

The electrochemical behavior of BN‐CNFs as anode materials for NIBs was investigated between a voltage window of 0–2.8 V. **Figure** **5**a shows the cyclic voltammetry (CV) curves of BN‐CNFs electrode for the first three cycles at a scan rate of 0.2 mV s^−1^. During the 1st discharge process, the large peaks at about 1.1 and 0.6 V, which disappear in the subsequent cycles, are attributed to the irreversible reaction between the Na‐ion and the surface functional groups,^45^ the decomposition of electrolyte, and the formation of solid electrolyte interphase (SEI) film.^46^ The peaks at about 0.01 V correspond to Na‐ion insertion in BN‐CNFs.^47^ The CV peaks located at about 0.01 V is ascribed to Na^+^ insertion/extraction in the interlayer of the BN‐CNFs.^47^, ^48^ A clear peak at 0.11 V in the 1st charge process is observed, corresponding to sodium extraction from the nanopores of BN‐CNFs.^11^ Remarkably, in the second and third cycles, the CV curves almost overlap, a long cathodic slope from 1 to 0.01 V and a broad anodic peak at 0.1V were observed, indicating the excellent reversibility of sodium insertion/extraction reaction.^2^, ^49^, ^50^, ^51^

**Figure 5:**
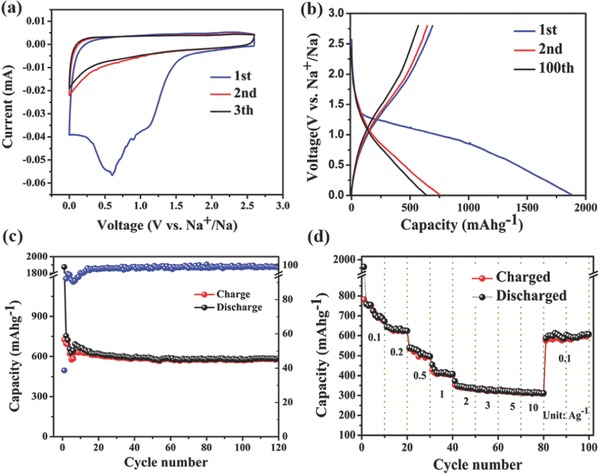
Electrochemical characterization of the BN‐CNFs electrode. a) Cyclic voltammetry curve at a scan rate of 0.1 mV s^−1^. b) Galvanostatic charge–discharge profile at a current density of 100 mA g^−1^. c) Cycling performance at a current density of 100 mA g^−1^. d) Rate performance at different current densities from 0.1 to 10 A g^−1^.

Figure 5b shows the voltage profiles of the BN‐CNFs at a current rate of 100 mA g^−1^. The capacities of the first discharge and charge are 1888 and 691 mAh g^−1^, respectively. The large irreversible capacity arises from both the decomposition of electrolyte at electrode surface to form the SEI films and the irreversible insertion of Na^+^ into unique pore structure.^38^ Figure 5c displays the cycling stability of the BN‐CNFs at 100 mA g^−1^. The BN‐CNFs show excellent cycle stability, and deliver a reversible charge capacity of 581 mAh g^−1^ after 120 cycles, corresponding to a capacity decay of 0.57% per cycle. Compare with other carbon‐based materials reported in literature, our sample shows improved reversible capacity, especially at low current density, demonstrating the reversible insertion of Na^+^ into the large interlayer space of carbon.^49^, ^52^, ^53^ In contrast, the CNFs, BN‐CNFs‐1, and BN‐CNFs‐2 electrodes show much lower reversible capacity and cyclability (Figure S4, Supporting Information). Figure 5d shows the rate capability of BN‐CNFs. It displays charge capacities of 644, 536, 428, 353, 329, 323, and 314 mAh g^−1^ at 0.2, 0.5, 1, 2, 3, 5, and 10 A g^−1^, respectively. After several cycles, the Coulombic efficiency approaches 100%. When the current density was tuned back to 0.1 A g^−1^, the reversible capacity of the BN‐CNFs could recover to 586 mAh g^−1^, exhibiting super rate performance. **Figure** **6**a shows the super long cycle performance at a high current density of 10 A g^−1^. Even after 1000 cycles, a comparable high reversible charge capacity of 277 mAh g^−1^ can still be obtained, showing capacity decay as low as 0.015% per cycle.

**Figure 6:**
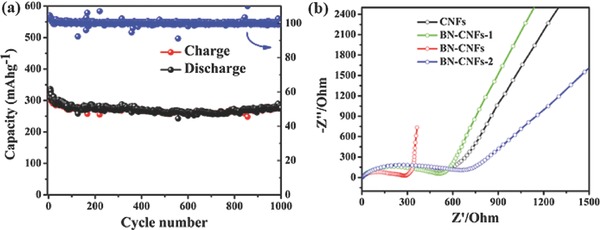
a) Cycling performance of BN‐CNFs at a high current density of 10 A g^−1^. b) Nyquist plots of CNFs, BN‐CNFs‐1, BN‐CNFs, and BN‐CNFs‐2 after 5 cycles at a current density of 100 mA g^−1^.

To further understand the improved sodium storage performance, we carried out the electrochemical impedance spectra of all samples. As shown in Figure 6b, the Nyquist plots possess a depressed semicircle in the high frequency and middle frequency regions and a straight line in the low‐frequency region. The SEI film, charge‐transfer process in the middle‐frequency and contact resistance at high‐frequency corresponds to the semicircle. And the straight line in the low‐frequency reflects Warburg impedance assigned to Na^+^ diffusion in the hard carbon.^11^, ^54^ Obviously, the BN‐CNFs show the lowest resistance, leading to the best sodium storage performance.

To better understand the synthetic chemistry of nitrogen and boron codoped carbon material as well as its adsorption ability of sodium atom, we performed density functional theory (DFT) calculations using the Vienna Ab Initio Simulation Package (VASP). We optimized different geometry configurations of boron and nitrogen doped graphene sheets,^55^ and calculated their formation energy as well as adsorption energy of Na atom, which is the key factor for the capacity of NIB. As illustrated in **Figure** **7**, the pristine calculation model is a 5 × 5 × 1 graphene supercell where nitrogen and boron atoms are doped inside the lattice. Even though there are some differences between our calculation models and experimental material of BN‐CNFs, it should not undermine the reliability for investigating the adsorption ability of carbon materials through simplified graphene models.^56^, ^57^ The formation energy (Δ*E*_f_) of the graphene with only boron doping model (B–gra) was calculated and compared to that with B and N codoping models (the B doping content is increasing from BN–gra to B_7_N–gra), the calculated doping energy of average B atom for each model is 1.10, 0.02, 0.39, 0.68, 0.85, 0.98, 1.05, and 1.14 eV, respectively (as illustrated in **Figure** **8**). It indicates that nitrogen doping can significantly decrease the formation energy of boron doped graphene. However, this down trend will decline and finally disappear with the increasing of B doping content (reaches its bottleneck when the doping content is about 12 at%). The calculated result illustrates possible formation chemistry of our BN‐CNFs materials, that is, under suitable experimental condition, B and N codoped material is more stable and accessible than those materials with only one kind of heteroatom. Interestingly, the highest level of B doping of our experimental material is about 10 at%, which is also very close to our calculation result 12 at%. In addition to the formation energy, we also calculate the adsorption energy of the pristine graphene and doped graphene to understand the interaction mechanism between Na and heteroatom. The optimized structures are illustrated in Figure 7 and the calculated adsorption energy (Δ*E*_a_) are listed in **Table** **1**.

**Figure 7:**
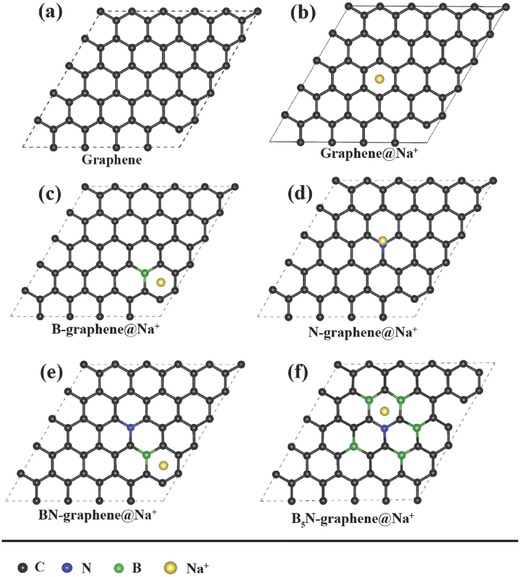
a) The optimized models of pure graphene. b–f) The optimized models of Na ion adsorbed on the models of pure graphene, B–graphene, N–graphene, BN–graphene, and B_5_N–graphene.

**Figure 8:**
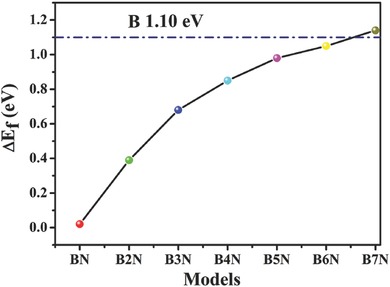
The formation energy (Δ*E*_f_) for average B atom doping of the models from BN to B_7_N.

**Table 1:** Adsorption energy of Na ion on different optimized structures

Structures	Graphene	N–gra	B–gra	BN–gra	B_5_N–gra
Δ*E*_a_ [eV]	−0.59	−0.22	−1.79	−0.91	−2.38

According to the calculated results, the adsorption energy of B doped graphene is −1.79 eV, which is about three times of pure graphene and nine times of N doped graphene, indicating B doping can significantly increase the Na adsorption ability of graphene than N doping. This result is also consistent with previous calculations.^58^ Besides, we also calculated the models with B and N codoping. The adsorption energy of BN–gra and B_5_N–gra are −0.91 and −2.38 eV, respectively, both of them are better than pure graphene and nitrogen doped graphene. Furthermore, the model with a higher level of B doping (B_5_N–gra) exhibits the better adsorption of Na, which is also consistent with our experimental result: the materials with higher B doping content also exhibits better electrochemical performance. From the adsorption geometry of B_5_N–gra, we can find that the doped B and N atoms will form a region which functions as a whole to adsorb Na, improving the adsorption ability of the graphene sheet. This calculated result also demonstrates the synergistic effect of N and B codoped graphene as NIBs, which explains the excellent performance of BN‐CNFs.

In summary, BN‐CNFs were synthesized through fully infiltrate NH_4_HB_4_O_7_·H_2_O into the BC pellicle followed by carbonization, exhibits an interconnected fibrous morphology. The obtained BN‐CNFs show superior electrochemical performance for Na storage, delivering a high specific charge capacity of 581 mAh g^−1^ at 100 mA g^−1^ after 120 cycles and a remarkable cycling stability (277 mAh g^−1^ at 10 A g^−1^ after 1000 cycles). Codoping carbons with both B and N atoms can create synergistic effects: enlarged carbon layer spacing for Na^+^ insertion, improved electrochemical activity, and electronic conductivity. The first‐principles DFT calculations were performed to understand the synergistic effect of N and B codoped carbon material. In addition, the 3D interconnected structure not only facilitates the Na^+^ migration, but also improves the structure stability of self‐support BN‐CNFs electrode. This strategy provides a facile and easy scale‐up way to design heteroatom doped carbon materials with excellent performance for sodium storage and also for other applications (i.e., lithium storage, supercapacitor, biosensing, catalysis, and gas adsorption).

## Experimental Section

*Materials*: The BC membranes were provided by Hainan Yeguo Foods Co, Ltd. All other chemicals were analytical grade and used as received without further purification.

*Preparation of BN‐CNFs*: The BC was immersed into aqueous solution of NH_4_HB_4_O_7_·H_2_O (0.1 m) for 24 h with slowly stirring at room temperature, then was freeze‐dried 24 h. The obtained sample was pyrolyzed at 2 °C min^−1^ to 500 °C for 1 h, and then at 5 °C min^−1^ to 1000 °C for 2 h in Ar atmosphere. After carbonization the products were extensively washed in hot deionized water for several times, and dried at 80 °C overnight. The concentration of NH_4_HB_4_O_7_ **·**H_2_O were varied to be 0, 0.02, 0.1, and 0.15 to control the degree of doping. The obtained products were denoted by CNFs, BN‐CNFs‐1, BN‐CNFs, and BN‐CNFs‐2, respectively.

*Characterization*: FESEM images were performed on a JSM‐6700 field emission scanning electron microscope (JEOL, Tokyo, Japan) at an acceleration voltage of 5 kV. HRTEM images were taken on a JEOL 4000EX transmission electron microscope (JEOL, Tokyo, Japan). XRD patterns were recorded on a Philips X'Pert PRO SUPER X‐ray diffractometer with Cu Kα radiation. XPS spectra were taken on an ESCA Lab MKII X‐ray photoelectron spectrometer using an Mg Kα radiation exciting source (1253.6 eV). Fourier transform infrared (FTIR) measurements were conducted on a Bruker Vector‐22 FTIR spectrometer over a range of 400–4000 cm^−1^. Raman spectra were taken on a Renishaw System 2000 spectrometer using the 514.5 nm line of Ar^+^ for excitation. The specific surface area was calculated at 77 K using BET method.

*Electrochemical Measurements*: The electrochemical performance of as‐obtained products were carried out using 2032 coin cells with Na metal(purity ≥99.5%, SCRC) as counter and reference electrode. The self‐supported as‐prepared samples were directly used as the working electrodes (Figure S5, Supporting Information). The typical area density of each electrode was about 1.5 mg cm^−1^. The electrolyte was 1.00 m NaClO_4_ in ethylene carbonate and diethyl carbonate (EC:DEC = 1:1 by wt.) with 5 wt% fluoroethylene carbonate additive, and glass fiber from Whatman was used as separator. The cells were assembled in an Ar‐filled glove box with moisture and oxygen content of less than 1 ppm. The galvanostatic charge–discharge tests were examined at a voltage of 0.01–2.8 V. Cyclic voltammetry measurements were conducted at a scan rate of 0.1 mV s^−1^ and electrochemical impedance measurements were taken in the frequency range from 100 kHz to 0.01 Hz with the voltage perturbation at ≈0.02 V on 1 CHI 660D electrochemical workstation (Chenhua Instrument Company, Shanghai, China) at room temperature.

*Theoretical Calculations*: DFT calculations were performed using the VASP, with supplied projector augmented wave potentials for core electrons.^59^, ^60^ The generalized gradient approximation of Perdew–Becke–Ernzerhof^61^ was used for the exchange‐correlation functional. The conjugate gradient algorithm was used in the structural optimization, providing a convergence of 10^−5^ eV in total energy and 0.01 eV Å^−1^ in Hellmann Feynman force on each atom. The atomic structures were fully relaxed in all calculations. To ensure the accuracy of the calculated results, the cutoff energy was set to 400 eV with a 7 × 7 × 1 K‐point mesh to represent the Brillouin zone. The formation energy was calculated through

(1) $$\Delta E_{f}=E_{{\mathrm{GN}}_{x}B_{y}}+y_{u_{c}}-(E_{{\mathrm{GN}}_{x}}+y_{u_{B}})$$

where $E_{{\mathrm{GN}}_{x}B_{y}}$ and $E_{{\mathrm{GN}}_{x}}$ are the energies of B and N codoped graphene and the pristine graphene (*x* = 0), respectively; *x* is the number of C atoms substituted by N atoms, *y* is the number of C atoms removed from the pristine graphene cluster after B doping, *u*_c_ is the total energy of perfect graphene per atom, *u*_B_ is the total energy of B crystal per atom. The adsorption (binding) energy of the sodium atoms was evaluated from

(2) $$\Delta E_{a}=[E_{{\mathrm{GN}}_{x}B_{y}{\mathrm{Na}}_{z}}-(E_{{\mathrm{GN}}_{x}B_{y}}+zE_{\mathrm{Na}})]/z$$

where $E_{{\mathrm{GN}}_{x}B_{y}{\mathrm{Na}}_{z}}$ and $E_{{\mathrm{GN}}_{x}B_{y}}$ are the energies of BN codoped graphene after Na adsorption and without adsorption, respectively.

## Supporting information

As a service to our authors and readers, this journal provides supporting information supplied by the authors. Such materials are peer reviewed and may be re‐organized for online delivery, but are not copy‐edited or typeset. Technical support issues arising from supporting information (other than missing files) should be addressed to the authors.

Supplementary
