# Supplementary material for: Sodium‐Ion Batteries: Improving the Rate Capability of 3D Interconnected Carbon Nanofibers Thin Film by Boron, Nitrogen Dual‐Doping
Source: Adv Sci (Weinh). 2017 Jan 20;4(4):1600468. doi: 10.1002/advs.201600468 (PMC5396155; doi:10.1002/advs.201600468)
Supplement: Supplementary file 1 — Supplementary [file ADVS-4-na-s001.pdf]

Copyright WILEY-VCH Verlag GmbH & Co. KGaA, 69469 Weinheim, Germany, 2017.

ADVANCED SCIENCE Open Access

Supporting Information

for *Adv. Sci.,* DOI: 10.1002/advs.201600468

Sodium-Ion Batteries: Improving the Rate Capability of 3D Interconnected Carbon Nanofibers Thin Film by Boron, Nitrogen Dual-Doping

*Min Wang, Yang Yang, Zhenzhong Yang, Lin Gu, Qianwang Chen, and Yan Yu**

Supporting Information

Sodium-ion Batteries: Improving the Rate Capability of Three-Dimensional (3D) Interconnected Carbon Nanofibers Thin Film by Boron, Nitrogen Dual-doping

Min Wang ,[a] Yang Yang ,[b] Zhenzhong Yang,[d,e] Lin Gu,[d,e] Qianwang Chen,[b] Yan Yu[*a,c]*

[a] CAS Key Laboratory of Materials for Energy Conversion, Department of Materials Science and Engineering, University of Science and Technology of China, Anhui Hefei 230026, China

[b] Hefei National Laboratory for Physical Science at Microscale, Department of Materials Science & Engineering, University of Science and Technology of China, Hefei 230026, China

[c] State Key Laboratory of Fire Science, University of Science and Technology of China, Hefei, Anhui, 230026, China

[d] Beijing National Laboratory for Condensed Matter Physics, The Institute of Physics, Chinese Academy of Sciences, Beijing 100190, China

[e] Collaborative Innovation Center of Quantum Matter , Beijing, 100190 , China

*Corresponding Author: Tel.: +86-(0)551-63607179

E-mail address: yanyumse@ustc.edu.cn (Yan Yu)

Table S1 atom ratio of C, N and B of CNFs, BN-CNFs-1, BN-CNFs and BN-CNFs-2.

Sample	C (atom %)	N (atom %)	B (atom %)
BN-CNFs-1	89.17	2.42	8.41
BN-CNFs	87.39	2.51	10.1
BN-CNFs-2	87.19	8.51	4.3

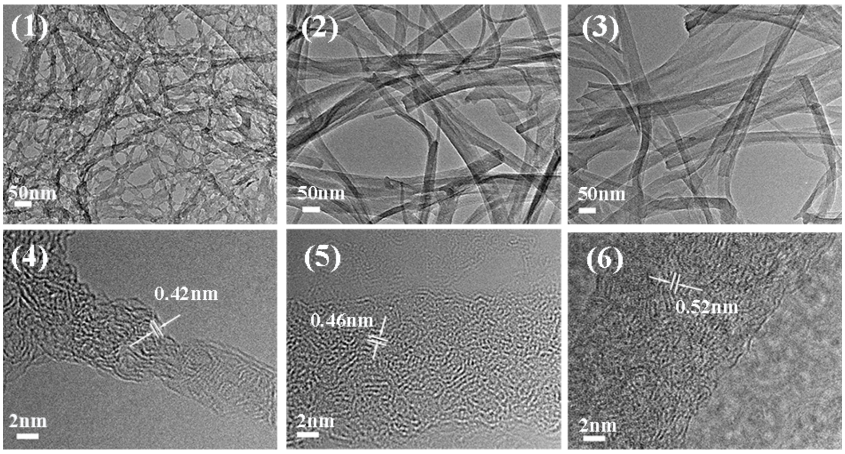

Figure S1 HRTEM images of CNFs, BN-CNFs-1 and BN-CNFs-2.

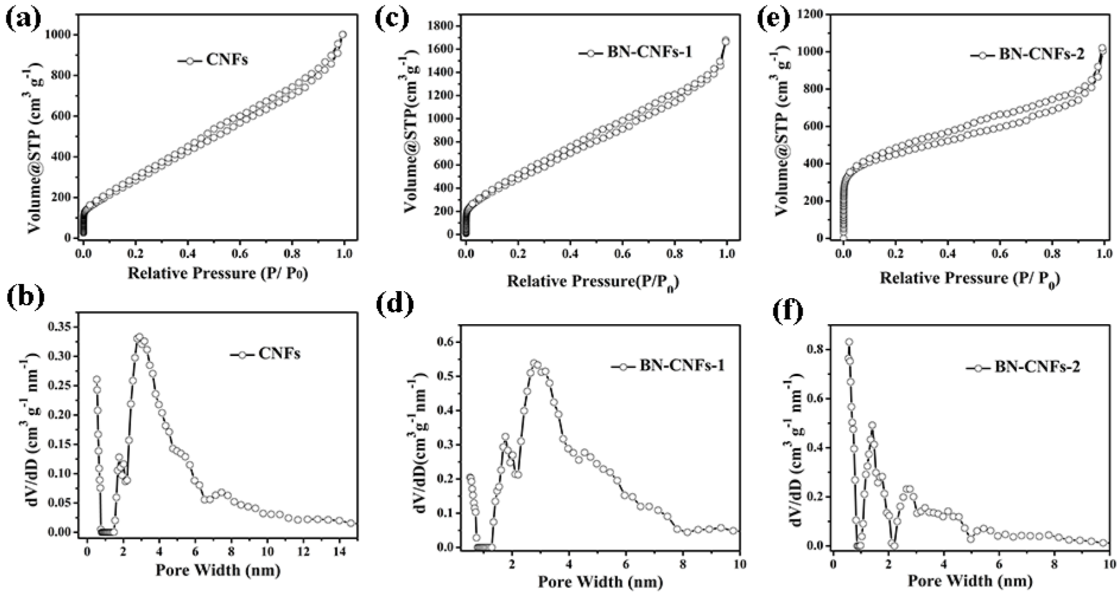

Figure S2 (a), (c) and (e) Nitrogen adsorption/desorption isotherms and (b),(d) and (f) the corresponding pore-size distribution curve from the adsorption isotherms using density functional theory (DFT) method of the CNFs, BN-CNFs-1 and BN-CNFs-2 composites.

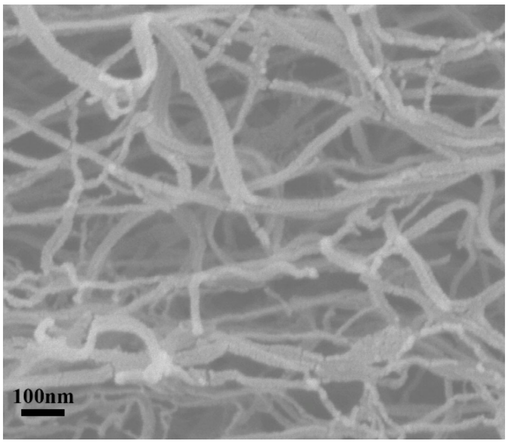

Figure S3 FESEM micrographs of BN-CNFs-2.

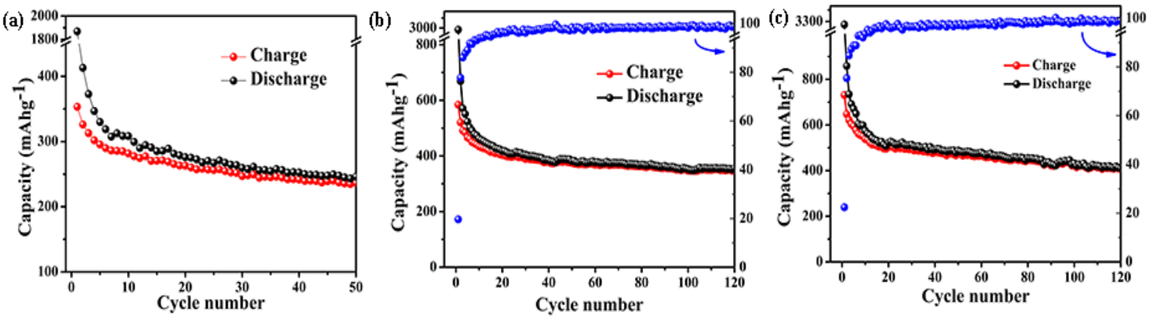

Figure S4 Cycling performance of CNFs, BN-CNFs-1 and BN-CNFs-2 samples at a high current density of 0.1 Ag^{-1}.

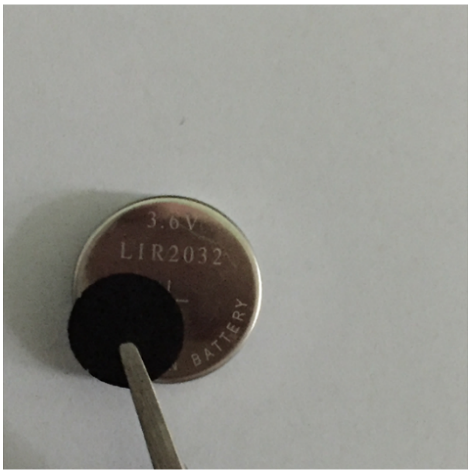

Figure S5 the digital image of BN-CNFs electrode.
